# Effects of Nrf2 deficiency on mitochondrial oxidative stress in aged skeletal muscle

**DOI:** 10.14814/phy2.13998

**Published:** 2019-02-12

**Authors:** Yu Kitaoka, Yuki Tamura, Kenya Takahashi, Kohei Takeda, Tohru Takemasa, Hideo Hatta

**Affiliations:** ^1^ Department of Human Sciences Kanagawa University Yokohama Japan; ^2^ Department of Exercise Physiology Nippon Sport Science University Tokyo Japan; ^3^ Department of Sports Sciences The University of Tokyo Tokyo Japan; ^4^ Graduate School of Comprehensive Human Science University of Tsukuba Tsukuba Japan

**Keywords:** Aging, mitochondria, oxidative stress, skeletal muscle

## Abstract

Oxidative stress and mitochondrial dysfunction are associated with the aging process. However, the role of nuclear factor erythroid 2 ‐related factor 2 (Nrf2) in skeletal muscle during aging remains to be clarified. In the current study, we assessed whether the lack of Nrf2, which is known as a master regulator of redox homeostasis, promotes age‐related mitochondrial dysfunction and muscle atrophy in skeletal muscle. Here, we demonstrated that mitochondrial 4‐hydroxynonenal and protein carbonyls, markers of oxidative stress, were robustly elevated in aged Nrf2 knockout (KO) mice because of the decreased expression of Nrf2‐target antioxidant genes. Mitochondrial respiration declined with aging; however, there was no difference between Nrf2 KO and age‐matched WT mice. Similarly, cytochrome c oxidase activity was lower in aged WT and Nrf2 KO mice compared with young WT mice. The expression of Mfn1 and Mfn2 mRNA was lower in aged Nrf2 KO muscle. Mitochondrial reactive oxygen species production per oxygen consumed was elevated in aged Nrf2 KO mice. There was no effect of Nrf2 KO on muscle mass normalized to body weight. These results suggest that Nrf2 deficiency exacerbates age‐related mitochondrial oxidative stress but does not affect the decline of respiratory function in skeletal muscle.

## Introduction

Aging is characterized by loss of skeletal muscle mass and function, a phenomenon known as sarcopenia. This loss of muscle function leads to increased risks of physical frailty and mortality (Cruz‐Jentoft et al. 2010). Therefore, understanding the mechanism of muscle atrophy with aging is important for identifying therapeutic targets and promoting health span. The aging process has considered to be driven by oxidative stress, originally proposed by Harman 1956 as the free radical theory. Given that the mitochondrial electron transport chain is a major source of reactive oxygen species (ROS), mitochondria are thought to play a crucial role in sarcopenia. In agreement with this hypothesis, many studies have shown increases in ROS production in aged skeletal muscle, associated with mitochondrial dysfunction and increased mitochondrial apoptotic susceptibility (Chabi et al. 2008; Dai et al. 2014). However, whether elevated ROS production is causally related to muscle atrophy with aging remains to be clarified.

Nuclear factor erythroid 2‐related factor 2 (Nrf2) is a transcription factor that has been regarded as the key regulator of antioxidant genes (Motohashi and Yamamoto 2004). In response to oxidative stress, Nrf2 translocates into the nucleus and binds to the antioxidant response element of its target antioxidant genes. We previously reported that Nrf2 deficiency aggravates denervation‐induced oxidative stress in skeletal muscle of young mice, while it has little effect on the loss of muscle mass (Kitaoka et al. 2016). However, effect of Nrf2 deficiency on antioxidant enzymes was reported to be greater in aged skeletal muscle than in the muscle of young animals (Miller et al. 2012). Intriguingly, ablation of Nrf2 results in impaired muscle regeneration in an age‐associated oxidative stress condition (Narasimhan et al. 2014). To explore the possible involvement of Nrf2 signaling in aging process, we assessed whether the lack of Nrf2 promotes age‐related mitochondrial oxidative stress and muscle atrophy in skeletal muscle.

## Methods

### Animals and experimental design

Nrf2 knockout (KO) mice were obtained from Jackson Laboratory (Bar Harbor, ME). Mice were genotyped by PCR analysis of tail DNA as previously reported (Kitaoka et al. 2016). Animals were group‐housed (3–4 per cage) in an air‐conditioned room on a 12:12‐h light–dark cycle with standard chow and water given ad libitum. Aged male (22 months old) Nrf2 KO mice and young (4 months old) and age‐matched (22 months old) male wild‐type C57BL/6J (WT) mice were used (*n* = 6–7 each group). Animals were euthanized by cervical dislocation, and muscles were quickly removed, snap‐frozen, and stored at −80°C. All experiments were approved by the Animal Experimental Committee of The University of Tokyo.

### RNA isolation and real‐time quantitative PCR

Gastrocnemius muscle was homogenized on ice in Trizol reagent (Life Technologies, Gaithersburg, MD), and then separated into organic and aqueous phases with chloroform. Total RNA was isolated using RNeasy Mini kit (Qiagen) from the aqueous phase following precipitation with ethanol. After RNA concentration was measured by spectrophotometry (Nanodrop ND1000, Thermo Scientific, Waltham, MA), first‐strand cDNA synthesis was performed using a high‐capacity cDNA reverse transcription kit (Applied Biosystems, Foster City, CA). The expression of Nrf2, Nqo1 (NAD(P)H quinone oxidoreductase 1), Cat (catalase), Gclc (glutamate‐cysteine ligase catalytic subunit), Sod1 (superoxide dismutase 1), Sod2, Fis1 (fission, mitochondrial 1), Drp1 (dynamin related protein 1), Mfn1 (mitofusin 1), Mfn2, Opa1 (optic atrophy 1) were quantified using the Thermal Cycler Dice Real‐Time System and SYBR Premix Ex taq II (Takara Bio, Shiga, Japan). All samples were run in duplicate. Tbp (TATA box binding protein) was used as a control housekeeping gene, the expression of which did not alter between groups. Forward and reverse primers for the aforementioned genes were shown in Table 1.

**Table 1:** Real‐time PCR primer sequences

Gene	Forward primer (5′‐3′)	Reverse primer (5′‐3′)
*Nrf2*	TTCTTTCAGCAGCATCCTCTCCAC	ACAGCCTTCAATAGTCCCGTCCAG
*Nqo1*	TTCTCTGGCCGATTCAGAGT	GGCTGCTTGGAGCAAAATAG
*Cat*	ACATGGTCTGGGACTTCTGG	CAAGTTTTTGATGCCCTGGT
*Gclc*	CAGTCAAGGACCGGCACAAG	CAAGAACATCGCCTCCATTCAG
*Sod1*	CCAGTGCAGGACCTCATTTT	TTGTTTCTCATGGACCACCA
*Sod2*	CCGAGGAGAAGTACCACGAG	GCTTGATAGCCTCCAGCAAC
*Fis1*	GCCTGGTTCGAAGCAAATAC	CACGGCCAGGTAGAAGACAT
*Drp1*	CGGTTCCCTAAACTTCACGA	GCACCATTTCATTTGTCACG
*Mfn1*	TTGCCACAAGCTGTGTTCGG	TCTAGGGACCTGAAAGATGGGC
*Mfn2*	GGGGCCTACATCCAAGAGAG	GCAGAACTTTGTCCCAGAGC
*Opa1*	GATGACACGCTCTCCAGTGAAG	CTCGGGGCTAACAGTACAACC

### Mitochondrial isolation

Mitochondrial fractions were isolated using differential centrifugation as previously described (Tamura et al. 2015). Briefly, quadriceps femoris muscles were mildly homogenized in mitochondrial isolation buffer (67 mm sucrose, 50 mm Tris, 50 mm KCl, 10 mm EDTA and 0.2% (w/v) fatty acid‐free bovine serum albumin, pH 7.4). The homogenate was centrifuged at 700*g* for 15 min at 4°C, and the supernatant was centrifuged for 20 min at 12,000*g*. The pellet was washed, and re‐suspended in mitochondrial isolation buffer. After the isolation procedure, the total protein content of samples was quantified using the bicinchoninic acid (BCA) protein assay (Pierce, Rockford, IL). Mitochondrial samples were used for analyses of 4‐HNE, protein carbonyl, mitochondrial respiration, and ROS production. We confirmed the purity of the mitochondrial fraction by Western blotting using antibodies against glyceraldehyde 3‐phosphate dehydrogenase (cytosolic marker) and cytochrome c oxidase (COX) subunit IV (mitochondrial marker; data not shown). In addition, the integrity of our mitochondrial isolation method was verified by adding exogenous cytochrome c in a separate experiment.

### Whole muscle lysate

Gastrocnemius muscle was homogenized in radioimmunoprecipitation assay (RIPA) buffer (25 mmol/L Tris‐HCl, pH 7.6, 150 mmol/L NaCl, 1% NP‐40, 1% sodium deoxycholate, and 0.1% sodium dodecyl sulfate [SDS]) supplemented with protease inhibitor mixture (Complete Mini, ETDA‐free, Roche Applied Science, Indianapolis, IN) and phosphatase inhibitor mixture (PhosSTOP, Roche Applied Science). The total protein content of samples was quantified using the BCA protein assay (Pierce).

### Western blotting

Equal amounts of protein were loaded onto 10‐% SDS‐PAGE gels and separated by electrophoresis. Proteins were transferred to polyvinylidene difluoride (PVDF) membranes, and western blotting was carried out using primary antibody of 4‐HNE (4‐hydroxynonenal; ab48506), Total OXPHOS Rodent WB Antibody Cocktail (ab110413), Fis1 (ab96764), Drp1 (ab56788), Mfn2 (ab124773) from Abcam (Cambridge, Mass., USA); Opa1 (#612606) from BD Transduction Laboratories (Tokyo, Japan). Ponceau staining was used to verify consistent loading. Blots were scanned and quantified using C‐Digit Blot Scanner (LI‐COR, Lincoln, NE).

### Protein carbonyl content

Protein carbonyl content was measured with a commercially available kit (#ROIK03; SHIMA Laboratories, Tokyo, Japan). After mitochondrial proteins were transferred to PVDF membrane as described above, the membrane was reacted with dinitrophenylhydrazine (DNPH) followed by Western blotting procedure.

### Enzyme activity

Tibialis anterior muscle was homogenized in 100 (v/w) of 100 mmol/L potassium phosphate buffer. Maximal activities of citrate synthase (CS) and COX were measured spectrophotometrically, following established protocols (Spinazzi et al. 2012). Catalase activity was determined using spectrophotometric method as previously described (Hadwan 2018). Total SOD (Mn‐SOD and Cu/Zn‐SOD) activity was determined using the Superoxide Dismutase Assay Kit (706002, Cayman, Ann Arbor, MI) following the manufacturer's instructions.

### Mitochondrial respiration

Freshly isolated mitochondria (60 *μ*g) were incubated in a reaction buffer (250 mmol/L sucrose, 10 mmol/L Tris base, 1 mmol/L MgCl_2_). Mitochondrial oxygen consumption was measured using Tecan Spark multi‐mode plate reader with MitoXpress Xtra fluorescent sensor reagent (Agilent Technology, Santa Clara, CA) to measure dissolved oxygen level (Ex: 380 nm/Em: 670 nm). Complex II‐driven state III respiration was stimulated by adding 10 mmol/L Succinate and 1 *μ*mol/L Rotenone and 2.5 mmol/L ADP. Relative fluorescent change per minute was calculated using operation software.

### Mitochondrial reactive oxygen species production

Freshly isolated mitochondria (20 *μ*g) were incubated in mitochondrial respiration buffer and 50 *μ*mol/L 2′7′ dichlorofluorescin (DCF). ROS emission was measured under state III respiratory condition through the addition of 10 mmol/L Succinate and 1 *μ*mol/L Rotenone, and 2.5 mmol/L ADP. Relative fluorescence change (Ex: 480 nm/ Em: 520 nm) was measured using a Tecan multimode plate reader.

### Statistical analysis

Data were expressed as mean ± standard error of mean (SEM). One‐way analysis of variance (ANOVA) was performed, followed by Bonferroni multiple‐comparison test (GraphPad Prism 6.0, La Jolla, CA). Statistical significance was defined as *P* < 0.05.

## Results

### Skeletal muscle mass and sarcopenic index

**Figure 1:**
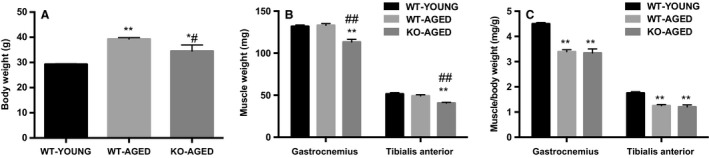
Body weight, skeletal muscle mass, and sarcopenic index in aged Nrf2 KO mice. (A) Body weight. (B) Gastrocnemius and tibialis anterior muscle mass. (C) Sarcopenic index (muscle mass per body weight). Data are presented as mean ± SEM. *n* = 6–7 in each group. *P < 0.05 **P < 0.01, significant difference versus WT‐YOUNG. ^#^ *P* < 0.05 ^##^ *P* < 0.01, significant difference versus WT‐AGED. WT, wild type; KO, knockout.

Animal characteristics are presented in Figure 1. Aged Nrf2 KO mice was lighter than aged WT mice (Fig. 1A). To investigate the effects of aging and Nrf2 deficiency on skeletal muscle mass, we measured the absolute mass of gastrocnemius and tibialis anterior muscles, and the sarcopenic indices (muscle mass per body weight). The absolute muscle mass was lower in aged Nrf2 KO mice compared with aged WT mice (Fig. 1B). Aging decreased sarcopenic index; however, there was no effect of Nrf2 deficiency (Fig. 1C).

### Antioxidant gene expression and oxidative stress

**Figure 2:**
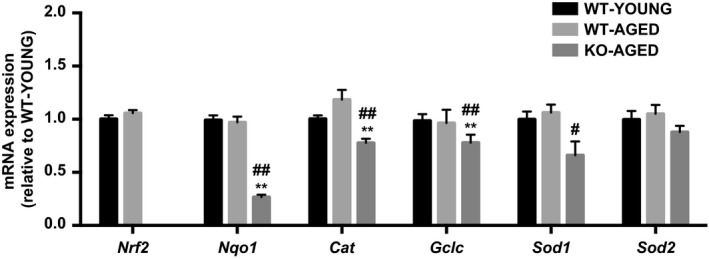
Nrf2 target antioxidant genes in aged Nrf2 KO mice. Data are presented as mean ± SEM *n* = 5–7 in each group. *P < 0.05 **P < 0.01, significant difference versus WT‐YOUNG. ^#^ *P* < 0.05 ^##^ *P* < 0.01, difference versus WT‐AGED. WT, wild type; KO, knockout.

**Figure 3:**
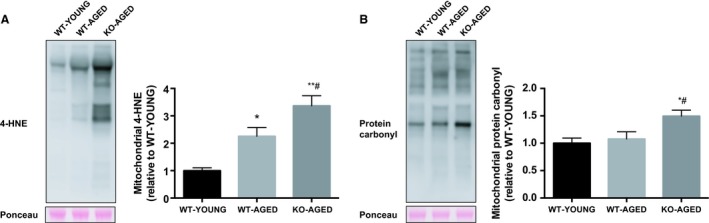
Mitochondrial oxidative stress in aged Nrf2 KO mice. A) Mitochondrial 4‐HNE content. B) Mitochondrial protein carbonyls. Data are presented as mean ± SEM *n* = 5–7 in each group. *P < 0.05 **P < 0.01, significant difference versus WT‐YOUNG. ^#^ *P* < 0.05, difference versus WT‐AGED. WT, wild type; KO, knockout.

**Figure 4:**
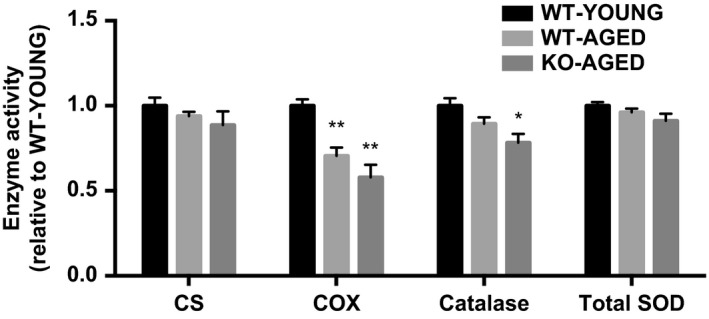
Enzyme activity in aged Nrf2 KO mice. Data are presented as mean ± SEM *n* = 5–7 in each group. *P < 0.05 **P < 0.01, significant difference versus WT‐YOUNG. WT, wild type; KO, knockout.

Knockout of Nrf2 was confirmed by undetectable mRNA expression compared to WT mice. There was no effect of aging on mRNA expression of Nrf2 and its major target genes (Nqo1, Cat, Gclc, Sod1, and Sod2). The expression of Nrf2 target antioxidant genes was decreased in aged Nrf2 KO muscle, except for Sod2, which was not altered (Fig. 2). To examine mitochondrial oxidative damage, we measured the level of 4‐HNE, a marker of lipid peroxidation, and protein carbonyl content, a marker of protein oxidation, in mitochondrial fractions. Nrf2 KO mice demonstrated substantial increases in 4‐HNE (Fig. 3A) and protein carbonyl content (Fig. 3B). Catalase activity was lower in Nrf2 KO mice compared with young WT mice, while total SOD activity was not altered (Fig. 4).

### Mitochondrial function and dynamics

**Figure 5:**
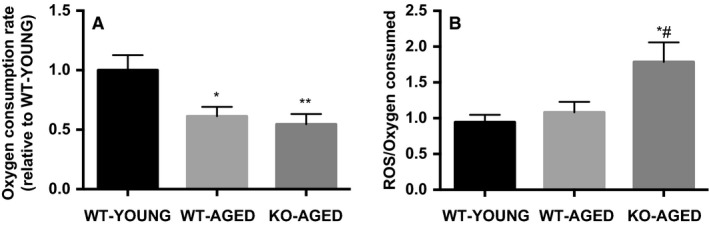
Mitochondrial function in aged Nrf2 KO mice. A) Oxygen consumption rate during mitochondrial respiration. B) Mitochondrial ROS production per oxygen consumed. Data are presented as mean ± SEM *n* = 5–7 in each group. *P < 0.05 **P < 0.01, significant difference versus WT‐YOUNG. ^#^ *P* < 0.05, difference versus WT‐AGED. WT, wild type; KO, knockout.

**Figure 6:**
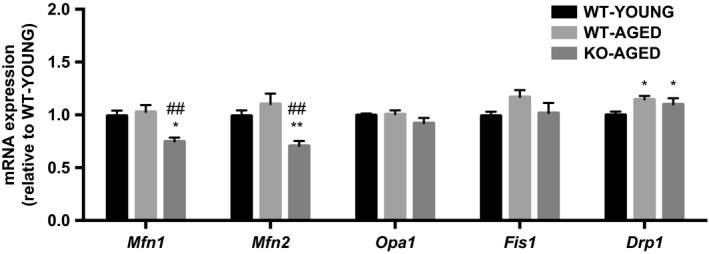
Mitochondrial dynamics genes in aged Nrf2 KO mice. Data are presented as mean ± SEM *n* = 5–7 in each group. *P < 0.05, significant difference versus WT‐YOUNG. ^##^ *P* < 0.01, difference versus WT‐AGED. WT, wild type; KO, knockout.

**Figure 7:**
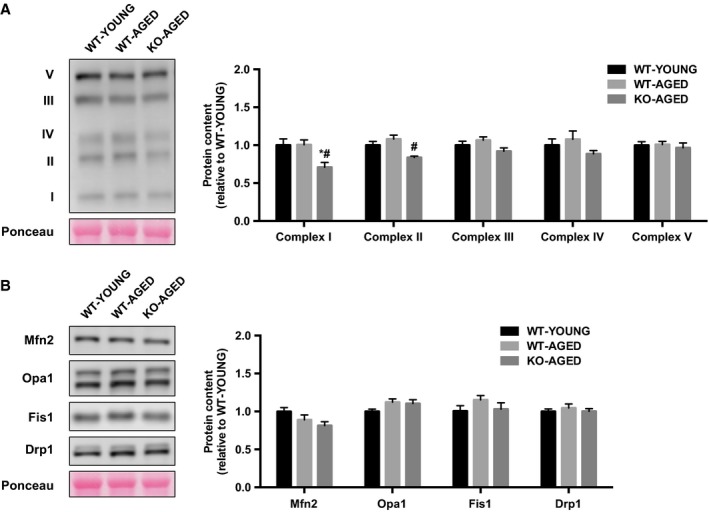
Mitochondrial protein levels in whole muscle lysate from aged Nrf2 KO mice. A) Mitochondrial OXPHOS proteins. B) Mitochondrial fusion and fission regulatory proteins. Data are presented as mean ± SEM *n* = 5–7 in each group. *P < 0.05, significant difference versus WT‐YOUNG. ^#^ *P* < 0.05, difference versus WT‐AGED. WT, wild type; KO, knockout.

To examine mitochondrial content, we first measured maximal activity of CS and COX, representatives of the TCA cycle and electron transport chain. COX activity was lower in aged WT and Nrf2 KO mice, whereas there was no difference in CS activity between groups (Fig. 4). Next, we measured mitochondrial respiration and ROS production as indicators of mitochondrial function. Mitochondrial respiration was decreased with age, while ROS production was higher in aged Nrf2 KO mice (Fig. 5A and B). To further assess mitochondrial quality, we evaluated mitochondrial dynamics regulatory gene expression. Drp1 mRNA was higher in aged muscle, while Mfn1 and Mfn2 mRNA were lower in aged Nrf2 KO mice (Fig. 6). At the protein level, Nrf2 KO resulted in decrease in mitochondrial complex I and II, while III, IV, and V remained unchanged (Fig. 7A). There was no effect of Nrf2 deficiency on mitochondrial fusion and fission proteins, although a decline in Mfn2 protein content approached significance (*P* = 0.10) (Fig. 7B).

## Discussion

Although mitochondria have been considered as a major potential mediator of sarcopenia, the molecular basis of the relationship between skeletal muscle mitochondria and sarcopenia is still unclear. Literature postulating an effect of aging on mitochondrial content has been controversial; however, the majority of reports showed increased ROS production with aging (Carter et al. 2015; Holloway et al. 2018). Given that mitochondria are not only the primal source of ROS but also the target of oxidative damage, ROS might create feedback loops, which exacerbate mitochondrial dysfunction. Previous studies investigated the relationship between Nrf2 and mitochondrial function (Holmstrom et al. 2013; Coleman et al. 2018). Loss of Nrf2 led to impaired mitochondrial respiration in mouse embryonic fibroblasts (Holmstrom et al. 2013) or muscle fibers of UCP1‐transgenic mice (Coleman et al. 2018); however, to the best of our knowledge, isolated mitochondrial fractions from aged Nrf2 KO skeletal muscle have not been examined. In this study, we found substantially increased oxidative stress in isolated mitochondrial fractions from aged Nrf2 KO muscle. As observed in earlier investigations, Nrf2 deficiency induced the decrease in the expression of its target antioxidant genes (Miller et al. 2012; Kitaoka et al. 2016). Importantly, previous studies demonstrated increases in ROS and 4‐HNE levels in whole muscle homogenate of aged Nrf2 KO mice (Miller et al. 2012; Narasimhan et al. 2014). The current study using isolated mitochondrial fractions indicated that the redox balance was altered toward accumulation of oxidative stress, which is regarded as an index of mitochondrial toxicity. Despite the elevated mitochondrial oxidative stress, however, there was no effect of Nrf2 deficiency on mitochondrial respiration and sarcopenic index in aged skeletal muscle. Lastly, we assessed mRNA and protein expression of genes related to mitochondrial fusion and fission, which is an important process in the maintenance of functional mitochondria (Yan et al. 2012). Morphologically, aging induces mitochondrial fragmentation or unusual enlargement in skeletal muscle (Iqbal et al. 2013; Leduc‐Gaudet et al. 2015). We observed that mitochondrial fusion regulatory genes were modestly down‐regulated in aged Nrf2 muscle, supporting that concept that ROS induces mitochondrial fragmentation (Fan et al. 2010; Iqbal and Hood 2014). However, at the protein level, the decline in Mfn2 with Nrf2 KO did not reach significance. Further study is needed to examine whether the mitochondria in aged Nrf2 KO muscle show aberrant ultrastructure by electron microscopy. Taken together, our results suggest that oxidative stress is not the proximate cause of muscle atrophy. Our data coincide with previous observations using Nrf2 KO mice in denervation‐induced muscle atrophy (Kitaoka et al. 2016) and streptozotocin‐induced diabetic atrophy model (Whitman et al. 2013).

Evidence supporting the role of mitochondrial oxidative damage in age‐related muscle dysfunction has been demonstrated using mice with genetically enhanced mitochondrial antioxidant activity (Umanskaya et al. 2014) or mice treated with mitochondrial protective peptide (Siegel et al. 2013). Furthermore, administration of sulforaphane, known as an Nrf2 activator, has shown improved muscle function in a mouse muscular dystrophy model (Sun et al. 2015). A limitation of this study is that we did not measure skeletal muscle fiber size/number and contractile function. Crilly et al. (2016) reported that Nrf2 KO mice demonstrated higher rate of fatigue in isolated muscle compared with WT animals. More recently, Ahn et al. (2018) reported that force generation normalized to muscle cross sectional area is reduced in old Nrf2 KO mice compared with age‐matched WT mice. These studies suggest that high levels of ROS exposure due to the absence of Nrf2 may alter muscle contractile function, not necessarily accompanied by changes in muscle mass.

In this study, we sought to examine the effect of Nrf2 deficiency on mitochondria in aged skeletal muscle. We demonstrated that Nrf2 deficiency enhanced mitochondrial ROS production in aged skeletal muscle and exacerbates age‐related oxidative stress, but has little effect on mitochondrial function or muscle mass.

## Conflict of Interest

There is no conflict of interest.
